# Editorial: Antimicrobials and Anticancers of Bacterial Origins

**DOI:** 10.3389/fmicb.2020.00842

**Published:** 2020-04-30

**Authors:** Ana R. Freitas, Tomasz M. Karpiński, Bingyun Li

**Affiliations:** ^1^UCIBIO/REQUIMTE, Departamento de Ciências Biológicas, Laboratório de Microbiologia, Faculdade de Farmácia, Universidade Do Porto, Porto, Portugal; ^2^Chair and Department of Medical Microbiology, Poznań University of Medical Sciences, Poznań, Poland; ^3^Department of Orthopaedics, School of Medicine, West Virginia University, Morgantown, WV, United States

**Keywords:** non-ribosomal peptide, bacterial toxin, anticancer activity, antimicrobial activity, antibiotic, bacteriocin, bacterial enzyme

Infectious diseases kill more than 17 million people a year according to the World Health Organization (WHO; https://www.who.int/whr/1996/media_centre/press_release). Among them, healthcare-associated infections caused by antimicrobial resistant (AMR) bacteria are increasingly hard to treat, threatening our progress in healthcare and life expectancy, and have a tremendous social and economic impact worldwide (https://www.who.int/news-room/fact-sheets/detail/antibiotic-resistance). In Europe alone, AMR causes 33,000 deaths and costs 1.5 billion per year in healthcare and productivity losses (EU Commission, 2017; Cassini et al., 2019). More than 2.8 million AMR infections occur in the United States each year, and more than 35,000 people die as a result (CDC, 2019). Patients with AMR infections may need to be hospitalized for more than 13 days, adding over 8 million hospital days annually (Ventola, 2015). Current strategies to counteract this worrying scenario include the investment on R&D to develop new antibiotics.

Cancer is another leading cause of morbidity and mortality worldwide; cancer was responsible for 8.8 million deaths in 2015. Similar to AMR infections, the resistance to classic cancer chemotherapeutic agents and/or novel targeted drugs has been recognized for decades and is a significant obstacle to the success of chemotherapy in cancer treatments.

It is clear that the greatest challenges in treating infections and cancers is the resistance to the treatments and the lack of new antimicrobial or anticancer drugs. Microorganisms themselves have been the richest source of antibiotics/anticancer drugs with currently unknown or unculturable bacteria being one of the greatest sources of novel bioactive molecules. Both antimicrobial and anticancer drugs can be obtained from bacteria of natural environments or the gut microbiota, and some drugs like actinomycin D and bleomycin may have dual antimicrobial and anticancer properties (Karpinski and Adamczak, 2018). The papers published in this Research Topic (seven Research Articles and three Reviews) have reinforced the diversity of bioactive molecules with antimicrobial and anticancer properties that can be found in natural bacteria, as illustrated further bellow.

Rodrigues et al. reviewed bacterial proteins and peptides called bacteriocins including colicin, pyocin, nisin, microcin, laterosporulin, pediocin, plantaricin, duramycin, etc. These unique proteins and peptides can be synthesized by Gram-positive (e.g., *Lactococcus lactis, Lactobacillus plantarum*) or Gram-negative (e.g., *Escherichia coli, Pseudomonas aeruginosa*) bacteria and were shown to present *in vitro* and/or *in vivo* antimicrobial and anticancer properties.

Baquero et al. comprehensively reviewed the main chemical substances arising from the action of the gut microbiota that might inhibit bacterial replication or reduce microbial viability. The role of microcins, which are small peptides produced by Gram-negative organisms (mostly *Enterobacteriaceae*) and are able to inhibit other bacteria, has been highlighted as effectors of intermicrobial interactions. The microcin's history and classification, mechanisms of action, and mechanisms involved in microcin's immunity (in microcin producers) and resistance (non-producers) were elegantly reviewed.

Shafiee et al. reviewed the targeted application of diphtheria toxin, one of the most frequently studied immunotoxins, in treating cancers. Strategies using cancer specific/targeted ligands and gene therapies were discussed.

In the study of Marcolefas et al. a variety of materials were collected from the high Arctic habitats, which are characterized by high salt and prolonged subzero temperatures, and analyzed for antimicrobial properties. Two Arctic microbial isolates presented antimicrobial activity either against foodborne pathogens at refrigeration temperatures or against clinically relevant pathogens (e.g., *Staphylococcus aureus, Enterococcus faecium, Acinetobacter baumannii*). This suggested that Arctic microbial diversity might be a promising source for antibiotics with unique low-temperature antimicrobial properties.

Qin et al. described a novel class I bacteriocin (Subtilin L-Q11) purified from a *Bacillus subtilis* strain L-Q11 of an orchard soil in Beijing, China. This novel bacteriocin showed optimal physicochemical features including high thermostability, pH-tolerance, resistance to chemical reagents, and sensitivity to various human proteases, and inhibition of growth of Gram-positive human pathogens (*Staphylococcus aureus* and *Enterococcus faecalis*) and food spoilage bacteria (e.g., *Bacillus cereus*). The most consistent activity of inhibition was observed against *Staphylococcus aureus*. Subtilin L-Q11 could be a potential antimicrobial drug for food preservation.

Zhao et al. reported a *Pseudomonas* sp. genome of a 11K1 strain isolated from rhizosphere in China by performing *in silico* and genetic analyses of putative secondary metabolite biosynthetic clusters. It was previously known that this strain exhibited strong inhibitory activity against plant pathogenic fungi and bacteria. Two putative novel cyclic lipopeptide biosynthetic clusters (brasmycin and braspeptin) were now identified to contribute to such antifungal activities by affecting biofilm formation and colony morphology.

The study by Cebrián et al. focused on the identification, design, and production of new lantibiotics, a class of polycyclic peptide antibiotics, with enhanced activity against *Clostridium difficile*. They used a strategy of genome mining, heterologous expression, design of new hybrid lantibiotics, and cloning and expressing the designed peptides in *Lactococcus lactis*. One peptide was found to present a high heterologous production level and high specificity/activity against clostridial species, making it a potential alternative to traditional antibiotics for treating *Clostridium difficile* infections.

Pérez Navarro et al. assessed the action of fluopsin C (a metalloantibiotic produced by *Pseudomonas* spp.) against multidrug-resistant and clinically-relevant bacterial species (*Staphylococcus aureus, Enterococcus faecium, Klebsiella pneumoniae*) through *in vitro* and *in vivo* experiments. Although further studies are needed to clarify the pharmokinetic and toxicity properties, fluopsin C and its derivatives have potential to serve as alternatives in treating multidrug-resistant infections caused by Gram-positive and Gram-negative bacterial species.

Can antimicrobial drugs be used for anticancer purposes or vice versa? Several antimicrobial drugs (e.g., antimicrobial peptides) were tested for their potential anticancer properties in the study of Varas et al.. For instance, the antimicrobial peptide microcin E492 produced by Gram-negative *Klebsiella pneumoniae* was found to present *in vitro* and *in vivo* anticancer properties against colorectal cancer cells. Models based on zebrafish were developed and may be efficient in screening pre-clinical anticancer drugs. Meanwhile, commonly used anticancer drugs (e.g., mitomycin C, etoposide, camptothecin, doxorubicin, 5-fluorouracil, cisplatin) were also repurposed for antimicrobial applications according to Domalaon et al. Mitomycin C, augmented with tobramycin-ciprofloxacin hybrids, was found to display antimicrobial activities against multidrug-resistant Gram-negative bacteria including *Pseudomonas aeruginosa, Acinetobacter baumannii, Escherichia coli, Klebsiella pneumoniae*, and *Enterobacter cloacae*. Therefore, there are drugs that can be used for both antimicrobial and anticancer purposes, and repurposing FDA approved drugs could be time- and cost-effective in discovering new antimicrobial or anticancer drugs.

Taken together, these studies illustrate the versatility of bioactive molecules from natural products and highlight the importance of using cutting-edge methodologies to boost the discovery of new antimicrobial and anticancer compounds.

## Author Contributions

All authors listed have made a substantial, direct and intellectual contribution to the work, and approved it for publication.

## Conflict of Interest

The authors declare that the research was conducted in the absence of any commercial or financial relationships that could be construed as a potential conflict of interest.
